# Gene hunting of the Genetic Analysis Workshop 16 rheumatoid arthritis data using rough set theory

**DOI:** 10.1186/1753-6561-3-s7-s126

**Published:** 2009-12-15

**Authors:** Chatchawit Aporntewan, David H Ballard, Ji Young Lee, Joon Sang Lee, Zheyang Wu, Hongyu Zhao

**Affiliations:** 1 Department of Psychiatry, Yale University, 300 George Street, Suite 503, New Haven, Connecticut 06511, USA; 2 Department of Mathematics, Chulalongkorn University, 254 Phayathai Road, Pathumwan, Bangkok, 10330, Thailand; 3 Program in Computational Biology and Bioinformatics, Yale University, 100 Howe Street, New Haven, Connecticut 06511, USA; 4 Biostatistics Resource, Keck Laboratory, Yale University, 300 George Street, Suite 503, New Haven, Connecticut 06511, USA; 5 Department of Epidemiology and Public Health, Yale University, 300 George Street, Suite 503, New Haven, Connecticut 06511, USA; 6 Department of Genetics, Yale University, 300 George Street, Suite 503, New Haven, Connecticut 06511, USA

## Abstract

We propose to use the rough set theory to identify genes affecting rheumatoid arthritis risk from the data collected by the North American Rheumatoid Arthritis Consortium. For each gene, we employ generalized dynamic reducts in the rough set theory to select a subset of single-nucleotide polymorphisms (SNPs) to represent the genetic information from this gene. We then group the study subjects into different clusters based on their genotype similarity at the selected markers. Statistical association between disease status and cluster membership is then studied to identify genes associated with rheumatoid arthritis. Based on our proposed approach, we are able to identify a number of statistically significant genes associated with rheumatoid arthritis. Aside from genes on chromosome 6, our identified genes include known disease-associated genes such as *PTPN22 *and *TRAF1*. In addition, our list contains other biologically plausible genes, such as *ADAM15 *and *AGPAT2*. Our findings suggest that *ADAM15 *and *AGPAT2 *may contribute to a genetic predisposition through abnormal angiogenesis and adipose tissue.

## Introduction

Rheumatoid arthritis (RA) is a chronic and systemic autoimmune disorder. It is characterized by synovitis - an inflammation of synovial membranes, which enclose joint. The afflicted joints become warm, swollen, tender, stiff, and in the final stage, deformed. RA is believed to be a heterogeneous disease because the manifestations are greatly varied across patients in terms of severity, progression, and response to therapy. It is estimated that genetic factors account for 60% of disease susceptibility [1]. The association between human leukocyte antigen (HLA) genes and RA has been well established, although HLA genes only account for 30% of the genetic contribution [1]. This distribution suggests that a genetic predisposition to RA involves non-HLA genes. There are many ongoing efforts to identify non-HLA genes associated with RA. Recent studies indicate that *PTPN22*, *OLIG3*/*TNFAIP3*, *STAT4*, and *TRAF1/C5 *genes may be involved in RA [2-5], and it is believed that many other genes are yet to be discovered.

To incorporate prior genome annotation information, we consider a gene-based analysis in this manuscript with the hope that a joint analysis of all the markers, e.g., single-nucleotide polymorphisms (SNPs), in a gene may increase statistical power for gene detection [6]. To achieve this goal, we employ the rough set theory (RST) [7], which is a method for feature selection to select informative SNPs for association analysis. Although a previous study concluded that RST did not identify disease-related loci [8], it only considered relative reducts, one of many options (e.g., global reducts, dynamic reducts, and generalized dynamic reducts) in RST for feature selection. The more robust definitions, such as generalized dynamic reducts, may offer higher power to detect genes associated with disease. We combined SNPs selected from RST to define joint patterns across these SNPs. We then clustered the joint patterns so that subjects sharing similar genotypes are grouped together. Statistical association was studied between disease status and cluster membership. In the following, we detail these steps and report findings from the analysis of North American Rheumatoid Arthritis Consortium (NARAC) data.

## Methods

The genome-wide RA data was collected by the NARAC and provided by the Genetic Analysis Workshop 16. SNPs with HWE *p*-value < 0.001 or MAF < 0.01 or with a missing percentage of an SNP or an individual > 0.1 were excluded. Subsequently, 500,884 SNPs from 1,140 controls and 862 cases remained. The remaining SNPs were assigned to their corresponding genes according to the gene annotation attached to the data. Note that the gene annotation assigned every SNP to a gene. The SNPs that were not in any gene regions were assigned to neighboring genes. In addition to case-control status, anti-cyclic citrullinated peptide (anti-CCP) and rheumatoid factor (RF) were used to classify clinical subgroups of RA patients. The subjects whose RF > 40 IU/ml were classified as RF+. A total of 1,961 subjects had anti-CCP data (1,330 anti-CCP+), and a total of 861 subjects had RF data (759 RF+).

We first give an overview of the RST [7]. Let *A *= (*U*, *A *∪ {*d*}) denote a decision table in which *U *is a set of subjects, *A *is a full set of SNPs in a specific gene, and *d *is a decision variable (case/control). An example of a decision table is shown in Table 1. A *reduct *is a minimal set of SNPs that are relevant to the decision variable. *B *⊂ *A *is a *relative reduct *if *B *is a minimal set such that ∂_*B *_= ∂_*A*_, where ∂_*B *_denotes a decision based on *B*. In Table 1, the universe *U *is {Subject1, ..., Subject6} and the decision *V*_*d *_is {Case, Control}. The function ∂_*B *_*U *→ *V*_*d *_is defined

as ∂ ↓ *B*(*x*) = {*v *∈ *V *↓ *d*: *x *might be *v *if we can observe only SNPs of *x *in *B*}. For example,

**Table 1:** An example of a decision table

Subject^a^	SNP1	SNP2	SNP3	Status
1	1	2	2	Case
2	2	1	2	Case
3	2	2	3	Case
4	1	2	1	Control
5	2	1	2	Control
6	1	2	3	Case

^a^The decision table contains all subjects in a study and all SNPs in a specific gene. SNP ∈ {1, 2, 3, missing}, where 1 = aa, 2 = aA, 3 = AA, Status ∈ {Case, Control}. This table was adapted from that in Pawlak [24].

whereas

Table 1 yields two relative reducts, {SNP1, SNP3} and {SNP2, SNP3}. The set of all relative reducts of decision table *A *is denoted by *RED*(A, *d*). Note that the above definition of relative reducts is also valid for a decision table with missing values.

The relative reducts are very sensitive to a small change in the decision table. Similar to the case in which a small prediction error is achieved without cross-validation, the prediction is not reliable because it overfits the data. *Dynamic reducts *provide a more robust solution to feature selection. It is defined as follows:

*DR*(**A**, **F**) denotes the set of all dynamic reducts of decision table **A**, and **F **denotes a finite set of subtables based on a subset of all the rows in the decision table. The default sizes of the subtables are 50%, 60%, 70%, 80%, and 90% [9]. For each size, ten subtables are randomly constructed. In each subtable, the ratio of cases to controls is approximately equal to that of the full table. A more relaxed definition is *generalized dynamic reducts*, denoted by *GDR*(**A**, **F**), where

The definition of GDR in Eq. (6) can be further loosened by equipping it with an epsilon,

where the stability coefficient (SC) is

*card*(**S**)denotes the cardinality of set **S**. The value of SC indicates the uniformity of a generalized dynamic reduct in the full decision table.

We set *ε *at 0.5 to discard the reducts that are statistically significant, but not stable (small SC). Among all the reducts found, we chose the one having the highest SC as a set of representative SNPs for a gene. The computational burden can be high when a gene contains hundreds of SNPs. Our empirical study showed that RST often selects among the top-ten SNPs ranked by an allelic test. To relieve the computational burden, for every gene we pre-selected 10 SNPs through allelic tests. There are 8,105 genes (49.01%) that contain more than 10 SNPs.

Starting from the top ten or fewer SNPs, RST yields a set of selected SNPs and their joint genotypes form a joint genotype pattern. We then applied the Pearson's chi-square test to calculate the *p*-value for genetic association based on this *n *× 2 table, where *n *is the number of distinct patterns, and the two columns correspond to cases and controls. The patterns with at least one missing SNP are excluded from the statistical test (no imputation). We use permutations to correct for multiple comparisons. For each permutation, the case and control status is shuffled, and the reducts are recomputed. We conducted 1,000 permutations to derive the empirical statistical significance level.

We used the complete-link clustering and agglomerative algorithm [10] to group individuals based on their joint genotype patterns. The optimal number of clusters was determined by the Dunn index [11]. The clustering algorithm was terminated when

where

where *k *denotes the number of clusters, ***P ***= {***X***_1_, ..., ***X***_*k*_} denotes a *k*-partition of ***X***, and ***X ***denotes the complete set of all items that we want to cluster. For instance, ***X ***= {***a***, ***b***, ***c***, ***d***, ***e***, ***f***}, ***k ***= 3 and ***P ***= {{***a***, ***b***, ***c***}, {***d***}, {***e***, ***f***}}The diameter of cluster ***A ***is **diam**(***A***), and the distance between clusters ***A ***and ***B ***is dist(***A***, ***B***). The distance between items *x *and *y*, *d*(*x*, *y*), is measured by mutual information (MI) [12]. MI clustering is motivated by the observation that subjects who shared similar haplotypes were put in the same cluster [13]. Another distance measure proposed in the literature is allele sharing (AS), which was used to classify HapMap populations [14]. We apply both MI and AS clustering, and then calculate the statistics of clustered patterns.

## Results

Based on the approach discussed above, we have identified non-HLA genes associated with RA (Table 2). These genes include some known genes, *TRAF1 *and *PTPN22*, and also include novel genes. In the following, we focus our discussion on two genes that are more biologically plausible, namely, *ADAM15 *and *AGPAT2*. Because the high degrees of freedom for these tables, we reduce the total number of patterns considered through clustering. The results are also shown in Table 2. MI clustering altered the ranks of *PTPN22*, *AGPAT2*, *ADAM15*, *TRAF1 *to three, four, five, and six, respectively, whereas the ranks altered by AS clustering were five, nine, seven, and six.

**Table 2:** Top-ranked genes associated with RA (22 autosomes, except chromosome 6)

Chr.	Gene symbol^a^	No. SNPs^b^	SC^c^	No clustering^d^		MI clustering		AS clustering	
						
				*p*-value	df^e^	*p*-value^f^	df^e^	*p*-value^f^	df^e^
16	*C16orf57*	2/2	1	6.06 × 10^-8^	5	6.06 × 10^-8 ^(1)	5	1.65 × 10^-2 ^(10)	3
21	*KRTAP10-6*	2/2	1	8.06 × 10^-6^	6	8.06 × 10^-6 ^(2)	6	4.42 × 10^-7 ^(1)	3
19	*DEDD2*	3/3	1	3.29 × 10^-5^	12	3.35 × 10^-3 ^(9)	2	1.84 × 10^-6 ^(2)	6
**9**	***AGPAT2***	**4/4**	**1**	**4.57 × 10^-5^**	**30**	**4.57 × 10^-5 ^(4)**	**5**	**3.30 × 10^-3 ^(9)**	**18**
**9**	***TRAF1***	**3/3**	**1**	**7.35 × 10^-5^**	**11**	**5.90 × 10^-5 ^(6)**	**2**	**8.91 × 10^-6 ^(6)**	**6**
15	*ODF3L1*	4/4	1	1.00 × 10^-4^	29	1.00 × 10^-4 ^(7)	29	1.89 × 10^-6 ^(3)	17
19	*RSHL1*	3/3	1	1.02 × 10^-4^	12	1.53 × 10^-3 ^(8)	1	1.09 × 10^-3 ^(8)	6
11	*CDC42BPG*	3/3	1	1.04 × 10^-4^	12	7.95 × 10^-1 ^(10)	1	2.55 × 10^-6 ^(4)	6
**1**	***ADAM15***	**7/7**	**1**	**1.08 × 10^-4^**	**64**	**5.62 × 10^-5 ^(5)**	**49**	**2.05 × 10^-5 ^(7)**	**40**
**1**	***PTPN22***	**6/7**	**0.5**	**1.24 × 10^-4^**	**34**	**1.52 × 10^-5 ^(3)**	**27**	**3.58 × 10^-6 ^(5)**	**20**

^a^The genes are sorted by *p*-value (no clustering). Only the top ten genes in this table were clustered.

^b^The number of selected SNPs and the total number of SNPs that were genotyped.

^c^SC, stability coefficient.

^d^The adjusted *p*-values (no clustering) for all genes in the table are less than 0.001 (1,000 permutations).

^e^df, degree of freedom, which is the number of clusters subtracted by one.

^f^The numbers in parenthesis are the ranks according to MI and AS clustering.

^g^Bold font indicates the genes that have been reported in the literature (*PTPN22 *and *TRAF1*) and the genes that we have found that are biologically plausible (*ADAM15 *and *AGPAT2*).

Because this data set is enriched for many gene signals on chromosome 6, all genes on chromosome 6 are excluded from Table 2, and they are shown separately in Table 3. Among HLA genes, *HLA-DRB1 *shows the strongest association to RA. *BTNL2 *seems to be the best candidate for non-HLA genes on this chromosome but the SC is relatively small, which implies that *BTNL2 *may only affect a subset of all RA cases.

**Table 3:** Top-ranked genes associated with RA on chromosome 6

Gene symbol	No. SNPs^a^	SC^b^	*p*-Value^c^
*HLA-DRB1*	6/6	1.00	4.89 × 10^-114^
*HLA-DRA*	10/10	0.90	1.18 × 10^-86^
*BTNL2*	10/10	0.36	6.86 × 10^-81^
*HLA-DQB1*	8/10	0.36	1.86 × 10^-77^
*HLA-DQA2*	10/10	1.00	6.19 × 10^-74^
*HLA-DQA1*	5/5	1.00	2.78 × 10^-72^
*C6orf10*	10/10	0.88	9.01 × 10^-70^
*LOC401252*	9/10	0.82	7.15 × 10^-51^
*HLA-DOB*	10/10	1.00	8.00 × 10^-32^

^a^The number of selected SNPs and the total number of SNPs that were genotyped.

^b^SC, stability coefficient.

^c^The adjusted *p*-values (no clustering) for all genes in the table are less than 0.001 (1,000 permutations). No clustering was performed.

To determine the RA subgroups associated with *BTNL2 *(SC = 0.36) and *PTPN22 *(SC = 0.54), we conducted association studies in two clinical subgroups classified by anti-CCP and RF (Table 4). Our analysis shows that *BTNL2 *was strongly associated with anti-CCP, but *BTNL2 *was not associated with RF (Table 4). *BTNL2 *is close to HLA genes, which are associated with the production of anti-CCP [15]. As a result, the association of *BTNL2 *with anti-CCP is apparently due to strong linkage disequilibrium with nearby HLA genes. There is probably no major role of *BTNL2 *in the pathogenesis of RA, as hypothesized by Orozco et al. [16]. Our calculations also indicate that *PTPN22 *was not associated with either anti-CCP or RF (Table 4).

**Table 4:** *BTNL2 *and *PTPN22 *loci associated with anti-CCP and RF

	*BTNL2 *gene		*PTPTN22 *gene	
		
Trait	anti-CCP	RF	anti-CCP	RF
SNPs selected/total no. SNPs genotyped	6/6	10/10	6/7	6/7
SC	1.00	1.00	1.00	0.88
*p*-Value	5.33 × 10^-166^	4.98 × 10^-1^	5.40 × 10^-2^	8.60 × 10^-1^
Adjusted *p*-value	< 0.001	0.560	0.026	0.870

## Discussion

Our findings indicate that, in addition to genes on chromosome 6, *PTPN22*, *TRAF1*, *ADAM15*, *AGPAT2*, and a number of other genes shown in Table 2 may be associated with RA. MI clustering increased the overall ranks of *PTPN22*, *AGPAT2*, *ADAM15*, and *TRAF1*. In contrast, AS clustering lowered the rank of *AGPAT2*.

MI clustering seems to perform better than AS clustering in association studies because MI exhibited clusters of similar haplotypes [13] but AS produced clusters of ancestral populations [14]. The allelic test based on single SNPs outside of chromosome 6 showed that *PTPN22*, *TRAF1*, and *ADAM15 *were in the top 25 genes (non-adjusted *p*-values: 1.21 × 10^-10^, 8.37 × 10^-8^, 2.00 × 10^-7 ^respectively) except for *AGPAT2 *(non-adjusted *p*-value: 1.69 × 10^-4^). As a result, *AGPAT2 *may have been overlooked in previous SNP-based analyses.

The genetic architecture of *ADAM15 *is noteworthy. The length of *ADAM15 *is one-third of the average human gene (the shortest of multiple-exon *ADAM *genes). It is comprised of 23 exons (2.5 times that of the human average) and 22 introns (the average length is less than one-tenth of the human average). At least 13 different splice variants have been found in normal human tissues [17]. These facts suggest that the identification of *ADAM15 *may be hindered due to its complex splicing.

The deterioration of arthritic joints is due to the fact that cells in synovial membranes that produce synovial fluids secrete several enzymes, collectively called matrix metalloproteinases (MMPs). MMPs are capable of degrading different types of extracellular matrix proteins, including bone and cartilage. A disintegrin and metalloproteinases (*ADAMs*) is a new family of metalloproteinases that shares structural similarities with MMPs as well as snake venom metalloproteinases. A high level of *ADAM15 *mRNA expression has been found in RA synovium [18]. As RA progresses, the synovial tissue expands and forms destructive panni - flaps of tissue hanging on synovium and particularly on cartilages. The tissue hyperplasia induces oxygen deficiency, which necessitates more blood vessels to supply oxygen. An amplification loop begins and therefore promotes angiogenesis, the formation of new blood vessels arises from pre-existing blood vessels. Because angiogenesis is a crucial step to progress bone damage, a blockade of angiogenesis would prevent the delivery of oxygen and nutrients to the inflammatory site. Inhibiting vascular endothelial growth factor (*VEGF*), which is a key factor in angiogenesis, showed a reduction in disease severity in animal models [19]. *ADAM15 *expression was up-regulated by *VEGF*, and linearly correlated with vascular density in synovial tissue [18]. A recent study suggested that *VEGF *and *ADAM15 *participate in an amplification loop. Inhibiting *ADAM15 *suppressed ocular neovascularization, a form of angiogenesis in eyes [20].

Although the mRNA expression level of *ADAM15 *was strongly associated with the progression and the severity of RA, the genetic predisposition to RA has not been elucidated yet. More association studies of *ADAM15 *in other independent populations, as well as better understanding of *ADAM15 *functions, are needed to confirm its effects in genetic predisposition to RA.

The involvement of *AGPAT2 *in RA is not straightforward. Generally, defects in *AGPAT2 *cause inherited lipodystrophy, a genetic disorder characterized by the selective loss of adipose tissue (fat loss) [21]. *AGPAT2 *is a crucial enzyme in biosynthesis of triacylglycerol, which is a primary form of fat stored in white fat cells. Thus, *AGPAT2 *is relevant to the formation of adipose tissue. White adipose tissue is ubiquitous in human joints. Although it used to be regarded simply as energy storage, it is now accepted that white adipose tissue is an endocrine organ that produces a variety of signaling proteins called adipokines. A significant level of three adipokines (leptin, adiponectin, resistin) has previously been found in synovial fluid of RA and osteoarthritis patients [22]. The adipokines play an important role in cross-talk between inflammatory system and adipocytes. It was concluded that the products of adipose tissue contribute to inflammatory and degenerative processes in common joint diseases [22]. Therefore, *AGPAT2 *may mediate inflammatory joints via adipose tissue.

The significant *p*-value of *TRAF1 *in Table 2 replicates the recent discovery of its association with predisposition to RA [5]. However, we could not replicate *STAT4 *and *OLIG3*/*TNFAIP3*'s association with RA (non-adjusted *p*-value = 0.04 and 0.02). Our failure to replicate *STAT4 *and *OLIG3*/*TNFAIP3 *may be due to the lack of a sufficiently large number of subjects as compared with the original study [3,4,23]. The other genes in Table 2 (*C16orf57*, *KRTAP10-6*, *DEDD2*, *ODF3L1*, *RSHL1*, and *CDC42BPG*) may contribute to RA, but no relevant literature suggests their potential role in the pathogenesis of RA.

We used the SNP annotation provided by Genetic Analysis Workshop 16 that assigns each SNP to one gene. There are 360,389 SNPs (67.78%) that are in gene deserts and are farther than 10,000 bp from the closest genes. A reviewer's suggestion is to define intergenic units and test them separately. Defining intergenic units may identify more genomic regions contributing to RA, but the results for *ADAM15 *and *AGPAT2 *still hold, because they contain only SNPs nearby (distance < 5,000 bp).

## Conclusion

RST may offer an effective alternative approach for SNP selection for genetic association studies. The stability coefficients can be used to filter out those genes that are statistically significant, but not stable. Consequently, this may help to increase our chance of distinguishing truly associated genes from those false signals. Our gene-based analysis has led to the identifications of a number of novel genes, with *ADAM15 *and *AGPAT2 *having plausible biological mechanisms related to development of RA. These two genes may contribute a genetic predisposition to an abnormality in angiogenesis and adipose tissue, which are important factors in the progression of RA.

## List of abbreviations used

anti-CCP: anti-cyclic citrullinated peptide; AS: Allele sharing; GDR: Generalized dynamic reduct; HLA: Human leukocyte antigen; MI: Mutual information; MMPs: Matrix metalloproteinases; NARAC: North American Rheumatoid Arthritis Consortium; RA: Rheumatoid arthritis; RF: Rheumatoid factor; RST: Rough set theory; SC: Stability coefficient; SNP: Single-nucleotide polymorphism; VEGF: Vascular endothelial growth factor.

## Competing interests

The authors declare that they have no competing interests.

## Authors' contributions

All authors participated in the design of the study. DHB and JYL performed data preprocessing. JSL and ZW performed statistical analysis. CA performed computer programming. HZ coordinated and helped to draft the manuscript. All authors read and approved the final manuscript.
